# Toward harmonization of aging and technology research: German adaptation of the mobile device proficiency questionnaire (MDPQ) for older adults

**DOI:** 10.1007/s10433-024-00834-w

**Published:** 2024-12-01

**Authors:** Anna Schlomann, Hans-Werner Wahl, Laura I. Schmidt, Nicole Memmer, Christian Rietz, Neil Charness, Walter R. Boot

**Affiliations:** 1 Network Aging Research, Heidelberg University, Bergheimer Straße 20, 69115 Heidelberg, Germany; 2 Institute for Educational Sciences, Heidelberg University of Education, Heidelberg, Germany; 3 Institute of Psychology, Heidelberg University, Heidelberg, Germany; 4 Division of Geriatrics and Palliative Medicine, Weill Cornell Medicine, New York, NY USA

**Keywords:** Digital literacy, Survey, Scale, German translation, Smartphone

## Abstract

The Mobile Device Proficiency Questionnaire (MDPQ) is a well-established, reliable, and valid instrument to measure basic and advanced mobile device skills among older adults. We offer a German adaptation of the MDPQ and report reliability and validity findings. A controlled translation procedure was applied. The translated scale was tested in two analytical samples with an age range of 50–95 years in 2020 (n = 548) and 2022 (n = 276). To assess reliabilities, internal consistencies and test–retest reliabilities are reported. To assess validity, we analyze the scale in the context of gender, age, and educational differences and its associations with measures of technology use and attitudes towards technology (convergent and divergent validity). The German adaptation of the MDPQ was found to be reliable and valid as the original version. The scale demonstrated an excellent internal consistency in both studies with α = 0.95 (study 2: α = 0.92) and ω = 0.95 (study 2: ω = 0.93). Subscale internal consistencies were all ≥ 0.65. Test–retest reliabilities with measurement waves 2 years apart showed excellent values (MDPQ full scale: r_tt_ = 0.84, *p* < 0.001). We also found the expected factorial structure of the scale, positive associations with education, mobile device use and technology attitudes, and negative associations with age. Women scored lower than men. The German adaptation of the MDPQ can serve as a useful tool to estimate mobile device skills in older adults in German speaking countries in a reliable and valid way, for example in survey research, research projects, and practice contexts. Translating and implementing the MDPQ in other European countries will help to further strengthen internationally harmonized assessments in technology and aging research.

**Supplementary Information:**

The online version contains supplementary material available at 10.1007/s10433-024-00834-w.

## Introduction

Digital skills are essential to participate actively in today’s societies for all ages including those in later life. In particular, Internet-connected mobile devices such as smartphones and tablets are increasingly important in various life domains such as communication, education, information search, health management, and leisure time (Charness and Boot 2022; Cotten 2017; Hunsaker and Hargittai 2018). Still, while information about ownership and frequency of use or non-use of mobile devices can be assessed quite easily, the assessment of digital skills is a more complex task. The ability to use digital devices according to one’s own preferences and goals is of critical importance for various life domains and its reliable assessment needs more investment in aging research.

### Technology proficiency as key factor for future quality of life in old age

The use of mobile devices among older adults has steadily increased over the last decades, but a digital divide between younger and older individuals still persists (Pew Research Center 2021; Rathgeb et al. 2021; Schlomann et al. 2020). In addition to this digital inequality of access or non-access (i.e., first level digital divide*)*, digital inequality related to digital skills has been conceptually described as second level digital divide (Scheerder et al. 2017; van Deursen and van Dijk 2019). Since older adults were less able to gain experiences with digital technologies during earlier stages of life (Sackmann and Winkler 2013), their digital skills, i.e., their technology proficiency, may also be lower compared to younger individuals’ technology proficiency and the question arises as how to measure this digital inequality related to the second level digital divide. For example, survey research monitoring historical-societal trends in technology proficiency across the full age range and following-up cohorts of older senior citizens is increasingly emerging across countries. Established reliable and valid assessments, ideally in an internationally harmonized way, is paramount for such survey work and its impact on society and policy. Further, at the micro to meso level of research, technology proficiency is on its way to become an important individual-difference variable in gerontology as well as a primary outcome in digitally oriented intervention research, implementation and education (Boot et al. 2015; Roque and Boot 2018, 2021).

### Assessment challenges of technology proficiency

An established standard of what high or low technology proficiency may mean is not established, although initiatives are emerging on the level of the EU such as the “DigComp 2.2” (Vuorikari et al. 2022) which aims at developing a common understanding and framework of digital competence in a broader population. Frequently applied 1-item self-ratings of digital skills (e.g., “How do you rate your competence in using mobile devices?”), are efficient and easy-to-use assessment methods, but also come with important limitations. First, it is difficult to estimate their psychometric properties; generally, reliability of 1-item measures is lower as compared to scales building on a set of pre-tested items. Second, 1-item approaches do not allow for any multi-dimensional understanding of technology proficiency, although it is likely that technology proficiency needs differentiation. Even if individuals already know how to perform basic functions in mobile device use, they may not be able to fully operate the devices due to their multi-functionality. Still, a basic proficiency is crucial for more sophisticated mobile device use and training success. Additionally, depending on an individual’s interest in using a mobile device, it might be used quite differently (e.g., entertainment, social purposes, information). Such multi-purpose use depending on individual’s priorities needs a broad-scale approach. In other words, 1-item approaches neither allow to distinguish between the skill levels for different purposes of usage nor to distinguish between basic or advanced usage purposes and skills. Third, reference points used to provide ratings of one’s own proficiency may differ considerably between individuals. For example, ratings may depend on one’s own more or less realistic/unrealistic expectations and attitudes related to digital technologies, views on technology communicated by others, or past experiences with technology at large. Individuals with relatively high level of technology proficiency might still rate their own competence as low when other persons in their social networks show even higher technology proficiency. To avoid these problems in estimating the full range of digital inequality related to the second level digital divide in societies, reliable and valid measures of proficiency with sufficient data representation of older adults are necessary.

### Existing scales to measure technology proficiency

While technology proficiency is generally important for an active participation in current societies, a distinction should be made between specific areas of proficiency. Different scales to measure technology proficiency have been proposed in this context. For example, Boot and colleagues (2015) proposed the Computer Proficiency Questionnaire (CPQ) and a short form of the CPQ (CPQ-12) to be able to measure computer proficiency focused on more traditional desktop applications in older adults (Boot et al. 2015). Recently, Roque and Boot (2021) also developed the Wireless Network Proficiency Questionnaire (WPQ) and its short form (WPQ-8) providing a proficiency measure for connecting to the Internet via wireless connection methods (Roque and Boot 2021).

In this paper, we focus on mobile devices such as smartphones and tablets. Due to the increasing distribution of mobile devices (Pew Research Center 2021) and particularly, the smartphone’s central role in setting up home networks, smartwatches, and other devices, as well as reduced offline services for more and more areas (e.g., transportation, banking), we argue that individuals without mobile device proficiency might be excluded from the significant advantages of these technologies for everyday life.

The Mobile Device Proficiency Questionnaire (MDPQ; 46 questions) and a short form of the MDPQ (MDPQ-16; 16 questions) (Roque and Boot 2018) are established measures to assess mobile device skills among older adults including eight subscales that cover basic and advanced proficiency: (1) mobile device basics, (2) communication, (3) data and file storage, (4) internet, (5) calendar, (6) entertainment, (7) privacy, and (8) troubleshooting and software management. Both scales, the MDPQ and the MDPQ-16 cover these eight subscales (3 to 9 items per subscale in MDPQ; 2 items per subscale in MDPQ-16). Based on the original MDPQ and MDPQ-16 scales in English language, adaptations were developed and tested in Spanish, Slovenian, Brazilian Portuguese, and Japanese (Moret-Tatay et al. 2019; Petrovcic et al. 2019; Raymundo et al. 2024; Shimokihara et al. 2024) but to date, no German adaptation exists. We expect that in the context of ongoing and increasing digital technology related research in German speaking countries (Germany, Switzerland, Austria, Luxembourg), the MDPQ may become an important tool to assess digital skills among (older) participants in a harmonized way as well as to serve practical needs such as to screen for those most in need for digital education and to facilitate individually-tailored technology training programs. Certainly, extending its use beyond German-speaking countries across Europe and beyond would be a definite goal for future research, if psychometric properties would unfold in sufficient quality across countries.

### Aims of the present study

The overall aim of this paper was to introduce a German version of the MDPQ-16, the *MDPQ-16G*. We put the focus on the short form of the MDPQ (i.e., MDPQ-16) since this is a parsimonious method to assess mobile device proficiency in a variety of research designs (e.g., large multi-topic survey studies, outcome assessment in controlled trials and practice contexts such as technology training programs, co-design workshops).

Our objectives were threefold: (1) We analyzed established reliability criteria of the MDPQ-16G, such as internal consistency and test–retest reliability. (2) We tested for convergent and divergent validity evidence of the scale. First, based on established previous findings related to current cohorts of older adults (König et al. 2018), we expect a significant negative age correlation as well as a significant positive association with education of the MDPQ-16G. Although gender differences in Internet access and use are declining (Bünning et al. 2023), we expect older women of current older cohorts to score lower than older men since they started to use the Internet somewhat later than men (Huxhold et al. 2020). Second, we analyzed the scale’s validity in the context of other measures relevant for digital proficiency. If the MDPQ-16G is assessing mobile device proficiency (rather than overall technological proficiency), divergent validity should be observed; it should be more related to self-reported mobile device use (i.e., smartphone, tablet) and less related to self-reported use of stationary devices (i.e., personal computer). Furthermore, we expect to observe convergent validity in the positive association of the MDPQ-16G with attitudes toward technology (i.e., technology acceptance, subjective technology adaptivity). (3) Taking advantage of the fact that we offered a paper–pencil as well as an online format as participating modes for our studies, we hypothesized that proficiency level is related to real-life decision making. We expected individuals who choose the online format to report higher levels of mobile device proficiency than those who choose the paper–pencil format.

## Method

### Participant recruitment and samples

We assessed the MDPQ-16G in two studies enrolled 2 years apart (2020 and 2022). In study 1 (June to September 2020), a sample of N = 548 individuals were recruited. Recruitment for study 1 was accomplished following two different approaches. First, we approached participants of a panel study focusing on subjective aging starting in 2012 (Diehl et al. 2013) that was enriched with technology-related measures in 2020. In total, n = 228 panel participants agreed to take part in study 1. Panel participants were given the choice to participate online or a via paper–pencil format served by mail. Second, we approached additional participants using existing lists of university programs for older adults at the universities of Frankfurt, Hannover, and Ulm. In total, n = 320 non-panel participants agreed to take part in study 1. Non-panel participants were only recruited online. All participants in study 1 were 50 years and older living in private households in Germany.

In study 2 (July to October 2022), we followed the same recruitment strategy. First, the same panel participants as for study 1 were approached. In total, n = 181 panel participants who already took part in study 1 were successfully recruited for study 2, resulting in two waves of measurement of the MDPQ-16G 2 years apart (T1–T2). Panel participants were again given the choice to participate online or a via paper–pencil format served by mail. This longitudinal sample with panel participants is only considered to analyze test–retest reliabilities. As our second independent sample for study 2, N = 276 individuals were recruited again via existing lists of university programs for older adults at the universities of Frankfurt, Hannover, and Ulm. None of these non-panel participants took part in the 2020 study. Again, all participants were 50 years and older living in private households in Germany.

Approval for the study was received from the Institutional Ethics Review Board of Heidelberg University’s Faculty of Behavioral and Cultural Studies (AZ Wahl 2020 1/1; AZ Wahl 2021 1/1). All participants gave written informed consent.

A summary of the participant demographics is provided in Table 1 for study 1 and in Table 2 for study 2. Participants’ mean age in study 1 and 2 ranged from 67.7 to 70.1 years. More women than men took part in the panel study. Among non-panel participants, more men than women took part. Education level was rather high in all samples. Altogether, the frequency of technology use was high with the highest frequencies of use for smartphones. Similarly, technology attitudes were rather positive (see Tables 1 and 2).

**Table 1:** Sample Characteristics in study 1 for panel and non-panel participants

	Participants Study 1		
	Panel	Non-panel	Total
N	228	320	548
*M* age (*SD*)	67.7 (9.64)	69.8 (6.83)	68.9 (8.18)
Age range	50–94	50–85	50–94
Gender			
% female	69.7	48.1	57.1
% male	30.3	51.9	42.9
Education			
% low	32.5	15.2	22.4
% high	67.5	84.8	77.6
Frequency of technology use			
* M* computer use (*SD*)	4.2 (2.18)	4.0 (2.29)	4.1 (2.24)
*M* smartphone use (*SD*)	5.0 (1.95)	5.5 (1.48)	5.3 (1.70)
*M* tablet use (*SD*)	3.1 (2.22)	3.7 (2.29)	3.4 (2.28)
Technology Acceptance (TAM)			
*M* usefulness (*SD*)	4.4 (0.79)	4.5 (0.69)	4.5 (0.74)
*M* ease of use (*SD*)	3.3 (0.88)	3.6 (0.91)	3.5 (0.91)
*M* Subjective technology adaptivity inventory (STAI) (*SD*)	3.1 (0.78)	3.4 (0.74)	3.3 (0.77)

*M*, Mean; *SD*, Standard Deviation; Education: low, no University entrance qualification/high, university entrance qualification; Frequency of technology use: 6-point scales from 1, never to 6, (almost) every day; Technology Acceptance (TAM): 5-point scales (1 to 5) with higher values indicating higher usefulness/ease of use; Subjective Technology Adaptivity Inventory (STAI): 5-point scales (1 to 5) with higher values indicating higher levels of subjective technology adaptivity

**Table 2:** Sample Characteristics in study 2 for panel and non-panel participants

	Participants Study 2	
	Panel	Non-panel
N	181	276
*M* age (SD)	68.7 (9.01)	70.1 (6.32)
Age range	52–95	56–90
Gender		
% female	68.5	39.1
% male	31.5	55.8
Education		
% low	33.1	10.7
% high	66.9	89.3
Frequency of technology use		
*M* computer use (*SD*)	3.9 (2.26)	3.8 (2.34)
*M* smartphone use (*SD*)	5.4 (1.57)	5.8 (0.94)
*M* tablet use (*SD*)	3.1 (2.19)	3.7 (2.31)
Technology Acceptance (TAM)		
*M* usefulness (*SD*)	4.1 (1.02)	4.5 (0.74)
*M* ease of use (*SD*)	3.3 (0.90)	3.6 (0.91)
*M* Subjective Technology Adaptivity Inventory (STAI) (SD)	–^a^	–^a^

*M*, Mean; *SD*, Standard Deviation; Education: low, no University entrance qualification/high, university entrance qualification; Frequency of technology use: 6-point scales from 1, never to 6, (almost) every day; Technology Acceptance (TAM): 5-point scales (1 to 5) with higher values indicating higher usefulness/ease of use

^a^Subjective Technology Adaptivity Inventory (STAI) only assessed in study 1

### Measures

#### MDPQ-16

For the construction of a German version of the MDPQ-16, a controlled translation procedure was applied. The translation process included the following steps according to the criteria described by Wild and colleagues (2005): As a first step, the forward translation of the English to German version was proposed by one bi-lingual speaker fluent in both English and German. After reconciliation, another expert in aging and technology research did a blind back-translation of the German version back to English. The back-translated version was discussed by other experts in aging and technology research who compared it to the original version (Wild et al. 2005). The final version of the MDPQ-16G translation was reconciled with all experts involved.

As in the original version, the MDPQ-16G is composed of 8 subscales that assess a broad range from basic to advanced mobile device proficiency: (1) mobile device basics, (2) communication, (3) data and file storage, (4) internet, (5) calendar, (6) entertainment, (7) privacy, and (8) troubleshooting and software management. Each subscale is composed of two items (i.e., 16 items in total). The items of the MDPQ-16G are scored on a 5-point scale like in the original version (1 = never tried, 2 = not at all, 3 = not very easily, 4 = somewhat easily, 5 = very easily). Answers given to the items of each subscale are averaged to get a measure for each subscale. The average scores of all subscales are then summed up across all subscales to calculate the total MDPQ-16G score (range 8–40) (Roque and Boot 2018). The full version of the MDPQ-16G is provided in Supplement A.

#### Additional analytical variables

(1) chronological age (continuous in years); (2) gender (dummy variable: male/female); (3) education level (dummy variable: low = no University entrance qualification/high = university entrance qualification); (4) general technology acceptance of modern technology: dimensions perceived usefulness (PU, mean of 3 items, range: 1–5, Cronbach’s α in study 1: α = . 89/study 2: α = 0.91) and perceived ease of use (PEOU, mean of 2 items, range 1–5, Spearman correlation in study 1: *r*_*S*_ = 0.71, *p* < 0.001/study 2: *r*_*S*_ = 0.71, *p* < 0.001) of Technology Acceptance Model (TAM; Davis et al. 1989). Higher values indicate higher perceived usefulness/higher perceived ease of use; (5) attitudes toward technology: Subjective Technology Adaptivity Inventory (STAI; Kamin and Lang 2013): scale that assesses the perceived personal adaptivity of technological environments. Higher values indicate higher levels of subjective technology adaptivity (mean of 12 items, range: 1–5, Cronbach’s α in study 1: α = 0.91^1^); (6) frequency of use (6-point scales, never to (almost) every day) of three devices (i.e., personal computer, smartphone, and tablet). Higher values indicate more frequent use; (7) assessment format for the subsample of panel participants (online format vs. paper–pencil format).

### Data analysis

The statistical analyses were conducted using IBM SPSS Statistics Version 27 and the lavaan package in R. We first provide the descriptive statistics of the MDPQ-16G and its subscales. Cronbach’s α (Cronbach 1951) as well as McDonald’s ω (Edwards et al. 2021) were reported to assess internal consistencies for the full MDPQ-16G scale due to pros and cons relevant for both coefficients (Revelle and Zinbarg 2009). We additionally report the Spearman-Brown reliability estimate and Spearman correlations for the MDPQ-16G subscales to better consider that sub-dimensions of the short MDPQ-16G solely rely on two items per dimension (Eisinga et al. 2013). Test–retest reliabilities were reported for panel participants who took part at both measurement waves 2 years apart (longitudinal sample).

To assess validity, we calculated t-tests for independent samples (online format vs. paper–pencil format; women vs. men; high vs. low education) and Pearson correlations (age, frequency of using digital devices, technology acceptance, technology adaptivity). To account for the interdependencies between gender and education, we additionally calculated a multiple regression analysis of the MDPQ-16G on individual characteristics.

The expected factorial structure of the MDPQ-16G was tested with a confirmatory factor analysis (CFA) with correlated factors. Given the ordinal scale of the questionnaire items, the Diagonally Weighted Least Squares (DWLS) estimator was used, as it is particularly suited for ordinal data and for cases where the assumption of multivariate normality may be violated (Mîndrilă 2010). Model fit was assessed using multiple fit indices, including the Chi-square statistic (χ^2^), the Comparative Fit Index (CFI), and the Root Mean Square Error of Approximation (RMSEA). To examine measurement invariance across gender and age groups, multi-group CFAs were conducted. For the age-based analysis, participants were divided into a middle-aged group (under 65 years) and an older age group (65 years and older). These analyses allow for testing whether the factor structure of the MDPQ-16 remains consistent across male and female participants, as well as across different age groups, thereby supporting the validity of the measure across gender and age categories.

## Results

### Reliability related findings

The overall level of the MDPQ-16G in our samples was relatively high with 30.0 (*SD* = 8.84) in study 1 and 34.5 (*SD* = 6.36) in study 2 (theoretical range: 8–40). For the subscales, the highest mean values (i.e., highest proficiency levels) were observed for “Mobile Device Basics”, “Communication”, and “Internet” in both independent samples (see Tables 3 and 4).

**Table 3:** MDPQ-16G scores and reliabilities in study 1 (total sample)

Scale	n	*M*	*SD*	*α*	ω	*r*_*SB*_	*r*_*S*_
MDPQ-16G	**544**	**30.0**	**8.84**	**0.95**	**0.95**	–	–
Mobile Device Basics	547	4.2	1.08	–	–	0.83	0.64
Communication	547	4.3	1.14	–	–	0.89	0.75
Data and File Storage	546	3.2	1.42	–	–	0.90	0.83
Internet	546	4.3	1.16	–	–	0.95	0.90
Calendar	546	3.5	1.70	–	–	0.97	0.93
Entertainment	544	3.3	1.58	–	–	0.80	0.68
Privacy	544	3.6	1.37	–	–	0.77	0.63
Troubleshooting and Software Management	544	3.5	1.55	–	–	0.94	0.88

MDPQ-16G, German short version of Mobile Device Proficiency Questionnaire; *M*, mean; *SD*, standard deviation; α, Cronbach´s α; ω, McDonald’s ω; r_SB_, Spearman-Brown reliability estimate; r_S_, Spearman correlations between two items of the subscales, all significant at *p* < .001; possible range of MDPQ-16G, 8–40; possible range of subscales, 1–5; α and ω can only be computed if the number of items is > 2. Values for the full MDPQ-16G scale are printed in bold

**Table 4:** MDPQ-16G scores and reliabilities in study 2 (non-panel participants)

Scale	n	*M*	*SD*	*α*	ω	*r*_*SB*_	*r*_*S*_
**MDPQ-16G**	**275**	**34.5**	**6.36**	**0.92**	**0.93**	**–**	**–**
Mobile Device Basics	275	4.5	0.81	–	–	0.70	0.51
Communication	275	4.8	0.63	–	–	0.88	0.63
Data and File Storage	275	4.0	1.11	–	–	0.86	0.83
Internet	275	4.6	0.82	–	–	0.92	0.84
Calendar	275	4.1	1.45	–	–	0.93	0.87
Entertainment	275	4.0	1.27	–	–	0.65	0.55
Privacy	275	4.3	1.00	–	–	0.71	0.60
Troubleshooting and Software Management	275	4.2	1.16	–	–	0.90	0.80

MDPQ-16G, German short version of Mobile Device Proficiency Questionnaire; *M*, mean; *SD*, standard deviation; α, Cronbach´s α; ω, McDonald’s ω; α and ω can only be computed if the number of items is > 2; r_SB_, Spearman-Brown reliability estimate; r_S_, Spearman correlations between two items of the subscales, all significant at *p* < 0.001; possible range of MDPQ-16G, 8–40; possible range of subscales, 1–5. Values for the full MDPQ-16G scale are printed in bold

The MDPQ-16G demonstrated an excellent internal consistency in both studies with α = 0.95 (study 2: α = 0.92) and ω = 0.95 (study 2: ω = 0.93). Subscale internal consistencies ranged from *r*_*SB*_ = 0.77 for the subscale “Privacy” (study 2: *r*_*SB*_ = 0.65 for the subscale “Entertainment”) to *r*_*SB*_ = 0.97 (study 2: *r*_*SB*_ = 0.93) for the subscale “Calendar” (see Table 3 and 4).

A full correlation table of all MDPQ-16G items is provided in Supplement B, Table I and II. Correlations between all items of the scale were all significant at *p* < 0.001. The range of correlations between the items that belong to the same subscale is between *r*_*S*_ = 0.63 for the subscale “Privacy” to *r*_*S*_ = 0.93 for the subscale “Calendar” (range in study 2: *r*_*S*_ = 0.51 “Mobile Device Basics” to *r*_*S*_ = 0.87 “Calendar”; see Tables 3 and 4).

A comparison of MDPQ-16G values for panel and non-panel participants in study 1 shows that non-panel participants score significantly higher than panel participants. However, internal consistencies and correlations between the items that belong to the same subscales are largely comparable between the two subsamples (see Supplement B, Table III).

In the longitudinal sample with panel participants only, the overall MDPQ-16G score was 28.2 (*SD* = 9.36) in study 1 and 29.7 (*SD* = 8.91) in study 2 which is a significant increase from study 1 to study 2 (*t*(178) = 3.98, *p* < 0.001). The MDPQ-16G demonstrated excellent test–retest reliability with r_tt=_ 0.84 (total scale) across the rather long retest time interval of 2 years (see Table 5). Test–retest reliabilities for the subscales also reached good to very good magnitudes (0.56 < *r*_*tt*_ < 0.77).

**Table 5:** Test–retest reliability of MDPQ-16G from T1 to T2 (panel participants)

Scale	n	*r*_*tt*_	*p*
MDPQ-16G	**179**	**0.84**	** < 0.001**
Mobile Device Basics	181	0.56	< 0.001
Communication	181	0.61	< 0.001
Data and File Storage	180	0.72	< 0.001
Internet	180	0.62	< 0.001
Calendar	180	0.75	< 0.001
Entertainment	179	0.72	< 0.001
Privacy	179	0.66	< 0.001
Troubleshooting and Software Management	179	0.77	< 0.001

MDPQ-16G, German short version of Mobile Device Proficiency Questionnaire; r_tt_, test–retest reliability measure; retest time interval: 2 years. Values for the full MDPQ-16G scale are printed in bold

We additionally calculated test–retest reliability of PEOU (*r*_*tt*_ = 0.58, *p* < 0.001) and PU (*r*_*tt*_ = 0.72, *p* < 0.001) as well as for technology use items (computer use: *r*_*tt*_ = 0.73, *p* < 0.001, smartphone use: *r*_*tt*_ = 0.72, *p* < 0.001, tablet use: *r*_*tt*_ = 0.74, *p* < 0.001) which also reached good to very good magnitudes.

### Validity related findings

#### Association with individual characteristics

The correlation of the MDPQ-16G with chronological age was *r*_*age*_ = − 0.29, *p* < 0.001 indicating a negative association in study 1. This could also be replicated in study 2 (*r*_*age*_ = − 0.30, *p* < 0.001). With a mean value of 28.3 (*SD* = 9.09) women scored significantly lower than men with medium effect size (*M* = 32.1, *SD* = 8.05; *t*(528) = 5.08, *p* < 0.001, *d* = 0.43) in study 1, but not in study 2 (women: *M* = 33.7, *SD* = 6.66; men: *M* = 35.1, *SD* = 6.13; *t*(259) = 1.73, *p* = 0.084, *d* = 0.22). In study 1, we observed a significant difference (*t*(538) = 2.63, *p* = 0.009, *d* = 0.27) in the level of the MDPQ-16G between participants with low education (*M* = 28.1, *SD* = 9.49) and with high education (*M* = 30.5, *SD* = 8.60) in the expected direction, although the effect size was small (study 2: low education level: *M* = 34.2, *SD* = 5.29; high education level: *M* = 34.6, *SD* = 6.51, *t*(259) = 1.24, *p* = 0.780, d = 0.06). A multiple linear regression (*F*(3, 526) = 30.45, *p* < 0.001, adjusted *R*^*2*^: 0.14) of the MDPQ-16G on age, gender, and education in study 1 revealed that the effects of female gender (*β* = − 0.24, *p* < 0.001) and age (*β* = − 0.31, *p* < 0.001) on the MDPQ-16G remained significant but not the effect of high education (*β* = 0.07, *p* = 0.081). The same pattern was observed in study 2 (*F*(3, 256) = 10.85, *p* < 0.001, adjusted *R*^*2*^: 0.10; *β*_*female*_ = − 0.14, *p* = 0.014; *β*_*age*_ = − 0.32, *p* < 0.001; *β*_*education*_ = 0.02, *p* = 0.791).

#### Association with technology device use and technology attitudes

As expected, higher correlations were observed between the MDPQ-16G and mobile device use (*r*_smartphone_ = 0.58, *p* < 0.001; *r*_tablet_ = 0.41, *p* < 0.001) than with personal computer use (*r*_computer_ = 0.08, *p* = 0.077). We observed positive correlations between the MDPQ-16G and the TAM dimensions PU and PEOU in study 1 (*r*_PU_ = 0.37, *p* < 0.001; *r*_PEOU_ = 0.46, *p* < 0.001) and in study 2 (*r*_PU_ = 0.31, *p* < 0.001; *r*_PEOU_ = 0.40, *p* < 0.001). The STAI was also positively correlated with the MDPQ-16G in study 1 (*r*_STAI_ = 0.54, *p* < 0.001).^2^

#### Association with data collection mode

The majority of panel participants (n = 181 versus n = 47) chose the online format instead of the paper–pencil format. As expected, significant differences in the MDPQ-16G scores between the two assessment formats were observed with large effect size (*t*(224) = 5.31, *p* < 0.001, *d* = 0.88): Participants who choose the online assessment format scored significantly higher (*M* = 29.2, *SD* = 8.81) compared to participants who choose the paper–pencil assessment format (*M* = 21.2, *SD* = 9.89).

Running the same analyses on validity related aspects separately for panel and non-panel participants largely revealed the same results (see Supplement B, Table IV) with one exception: Mobile device proficiency in participants with low vs. high education differed neither in the panel subsample nor in the non-panel subsample.

#### Factor structure of the MDPQ-16G

The 16 items of the MDPQ-16G were expected to represent eight different domains of mobile device proficiency. A CFA with participants in study 1 supported the hypothesized eight-dimensional structure of the German adaptation of the MDPQ-16, with an excellent model fit (*χ*^*2*^ = 27.71, df = 76, *p* = 1.00; CFI = 1.00; RMSEA = 0.00). Table 6 presents the factor loadings for each item within the eight-dimensional model.

**Table 6:** Confirmatory factor analysis of MDPQ16-G (total sample in study 1)

Factors/MDPQ-16G Subscales	MDPQ-16G Items	Factor loadings
1: Mobile Device Basics	1a: Navigate using touchscreen	0.89
	1b: Use keyboard	0.79
2: Communication	2a: Send emails	0.86
	2b: Send pictures by email	0.93
3: Data and File Storage	3a: Transfer to computer	0.94
	3b: Transfer from computer	0.88
4: Internet	4a: Find information about hobbies	0.97
	4b: Find health information	0.92
5: Calendar	5a: Enter events into calendar	0.99
	5b: Check calendar	0.95
6: Entertainment	6a: Find entertainment apps	0.82
	6b: Listen to music	0.81
7: Privacy	7a: Setup password	0.79
	7b: Erase temporary files	0.79
8: Troubleshooting and Software Management	8a: Update apps	0.93
	8b: Delete apps	0.97

MDPQ-16G, German short version of Mobile Device Proficiency Questionnaire; Confirmatory Factor Analysis with Diagonally Weighted Least Squares estimator; Model fit: *χ*^*2*^, 27.71, df, 76, *p*, 1.00; CFI, 1.00; RMSEA, 0.00

To examine measurement invariance across gender and age groups, multi-group CFAs were conducted. Results indicated that both models achieved metric invariance, showing strong consistency across male and female as well as across middle-aged and older participants. The fit indices for both models were also excellent (gender: *χ*^*2*^ = 46.51, df = 160, *p* = 1.00; CFI = 1.00; RMSEA = 0.00; age groups: *χ*^*2*^ = 35.16, df = 160, *p* = 1.00; CFI = 1.00; RMSEA = 0.00), suggesting that the MDPQ-16G captures mobile device proficiency in a comparable way in men and women and among different age groups of middle-aged and older adults.

## Discussion

To understand barriers and facilitators of technology adoption among older adults remains a highly important research topic because digital participation is associated with participation in everyday activities and societal participation at large. In this context, inequalities in technology proficiency may be a more relevant indicator of digital inequality than the question of access or non-access alone because the full potential of the technology only unfolds when a person has the skills to handle and use it according to their preferences. A reliable and valid assessment of technology proficiency may also be an important screening tool for those in particular need for digital education and training programs.

Against this background, this paper aimed to introduce a German adaptation of a measure of technology proficiency to be used in a variety of research and practice contexts, the short form of the Mobile Device Proficiency Questionnaire (MDPQ-16). Our analyses showed that the German adaptation of MDPQ-16, the MDPQ-16G, is reliable and valid as the original English version.

A systematic review on digital literacy among older adults (Oh et al. 2021) summarizes that the MDPQ is the only available measure that covers all elements of five areas of digital literacy defined in a framework by the EU (i.e., Digi-Comp framework), which underlines the importance of a German adaptation of the scale and its relevance for the European context. Since established English, Spanish, Slovene, Brazilian Portuguese, and Japanese versions already exist, intercultural assessments of technology proficiency will be possible from a European and an international comparative research perspective and aging and technology researchers from other European countries may be encouraged to translate and implement the scale and by this means strengthen internationally harmonized assessments in digital aging contexts.

Internal consistencies of the MDPQ-16G were on very good levels. Our results show very similar findings on the overall reliability of the scale (original MDPQ-16 scale: α = 0.96, see Roque and Boot 2018; MDPQ-16G: α = 0.95 in study 1/α = 0.92 in study 2). Internal consistencies of the MDPQ-16G subscales were on average slightly lower in our studies, but still on a good to acceptable level. The relatively lower levels of internal consistency of some subscales (e.g., subscale privacy) may be attributed to the varying levels of item difficulty within these subscales, which can lead to reduced item correlations. Still, confirmatory factor analysis confirmed the eight-factor structure of the MDPQ-16G, demonstrating good model fit and supporting the theoretical domains.

The scale also showed excellent test–retest reliabilities in two survey waves 2 years apart. The long time interval between the two assessments is an important distinguishing feature of our study and the results underline the stability of the measurement. However, due to the already high level of mobile device proficiency in study 1, no large increases in the proficiency level were possible in our sample which may be an additional reason for the high measurement stability.

Our analyses of the validity criteria showed a negative correlation of the MDPQ-16G with chronological age which is an indication for a persisting age-related digital divide. Furthermore, women, and participants with a low education level scored lower on the MDPQ-16G than men and participants with a high education level. At the same time, we also found evidence that the digital gap may be closing in our analyzed group of highly educated participants since the observed differences were generally small and partially vanished in study 2. Our study participants reported a rather high frequency of technology use and a high level of mobile device proficiency at large, which even increased over the 2-year observation period from 2020 to 2022. This trend may be attributed to the fact that many older adults increased their use of mobile devices during the COVID-19 pandemic, which likely contributed to the enhancement of their technological skills over time. Compared to this, mobile device proficiency levels were lower in the original MDPQ-16 study (Roque and Boot 2018). The German SIM study (Rathgeb et al. 2021) furthermore shows that currently, only one fifth (22%) of people aged 60 years and older rate their competence to use a smartphone as good or very good and only 17% rate their competence to use a tablet as good or very good. The high technology affinity in our sample is further supported by the observation that most panel participants, given the choice between an online and a paper–pencil format, chose the online assessment format. Still, in the comparison of the paper–pencil vs. online assessment format, we observed significantly lower levels of mobile device proficiency among participants in the paper–pencil format. While this is also another proof of validity of the measure, this may also speak to ceiling effects of the MDPQ-16G for participants in the online assessment format which were previously observed among younger age groups in the construction of the original English scale as well as in the Spanish adaptation (Moret-Tatay et al. 2019; Roque and Boot 2018).

As a test of convergent and divergent validity, we expected mobile device proficiency to be higher correlated with mobile device use than with (stationary) computer use and to be positively correlated with technology acceptance and other technology attitudes such as the TAM and the STAI. These assumptions also hold true after our analyses indicating that the German adaptation of the MDPQ-16 met all of the analyzed reliability and validity criteria.

### Practical implications

The MDPQ-16G can be a powerful tool in survey research, research projects, and practice contexts. In survey research, the German translation of the scale contributes to the goal of an internationally harmonized technology and aging research and translations in other languages may follow. In the future, the MDPQ could then also be implemented in large European survey studies such as the Survey of Health, Ageing and Retirement in Europe (SHARE-ERIC 2024). In practice contexts, the MDPQ-16G can be a helpful screening tool to identify individuals in particular need of digital education and training programs. Assessing the MDPQ-16G prior to technology training courses can help to group individuals with similar skills into a course and to tailor the content and the speed of the program to the learners.

### Limitations

As in all research, this study was subject to limitations. The sample was not representative for older adults and participants with a high education level and high technology affinity were overrepresented meaning that proficiency level for the general population is overestimated. Differences between individuals with high vs. low education could only be observed to a limited extent. Furthermore, we could only test the measure in a sample with limited age range starting at an age of 50 years and we were not able to test the scale in a young age comparison group. Replication studies in more representative samples and among participants with a larger age range should be performed to assess the stability of results and to aim for country-level comparisons. Other aspects of limitation are related to the measure itself: First, the MDPQ-16G is a self-report proficiency scale and does not measure objective proficiency based on accomplishing tasks with mobile devices. Second, due to the large scope of data collection in the survey, we were only able to test the short version of the MDPQ with 2-item ratings for each subscale. For the same reason, the TAM could only be assessed with a limited number of items. This small number of items provides a less reliable estimation of the constructs than corresponding scales with more items. This should be addressed in future studies. Still, confirmatory factor analysis confirmed the eight factorial structure as theoretically expected. Third, although the MDPQ-16G covers a broad range from basic to advanced mobile device usage and skills, some specific forms of usage remain unconsidered. Further developments might for example include more recent forms of communication such as video calls, social media use or consulting with digital assistants.

## Conclusion

The German adaptation of the MDPQ is available for implementation in German speaking research and practice contexts. It can serve as a useful tool to better estimate mobile device skills among German speaking older adults in a reliable, valid, and internationally harmonized way.

## Supplementary Information

Below is the link to the electronic supplementary material.

Supplementary file1 (PDF 252 kb)

Supplementary file2 (PDF 427 kb)

## Data Availability

The raw data supporting the conclusions of this article will be made available by the authors, without undue reservation.

## Footnotes

1 The STAI was only assessed in study 1.

2 The STAI was only assessed in study 1.
