# Supplementary material for: Toward harmonization of aging and technology research: German adaptation of the mobile device proficiency questionnaire (MDPQ) for older adults
Source: Eur J Ageing. 2024 Dec 1;21(1):38. doi: 10.1007/s10433-024-00834-w (PMC11609131; doi:10.1007/s10433-024-00834-w)
Supplement: Supplementary file 1 — Supplementary file1 (PDF 252 kb) [file 10433_2024_834_MOESM1_ESM.pdf]

Supplement A

German Translation of Mobile Device Proficiency Questionnaire (MDPQ-16G)

Dieser Fragebogen fragt nach Ihren Fähigkeiten, eine Reihe von Aufgaben mit einem **mobilen Endgerät** durchzuführen.

Was ist ein mobiles Endgerät?

Ein mobiles Endgerät ist ein Gerät, das es Ihnen ermöglicht, die meisten Aufgaben wie an einem Computer durchzuzuführen, jedoch ohne Tastatur und Maus. Stattdessen verwenden diese Geräte einen Touchscreen als Schnittstelle zwischen dem Benutzer und Programmen (genannt Apps - kurz für Applications, also Anwendungen).

Bitte beantworten Sie jede Frage, indem Sie ein X in das Feld setzen, das am ehesten auf Sie zutrifft.

Wenn Sie bisher noch nie versucht haben, eine Aufgabe durchzuführen oder nicht wissen, was es ist, markieren Sie bitte "**NOCH NIE PROBIERT**", unabhängig davon, ob Sie denken, dass Sie in der Lage sind, die Aufgabe auszuführen. **Bitte denken Sie daran, dass Sie Ihre Fähigkeit bewerten, jede dieser Aufgaben speziell mit einem mobilen Gerät (Tablet-Computer oder Smartphone) auszuführen.**

1. Grundlagen mobiler Geräte

Bei der Nutzung eines mobilen Endgeräts kann ich:	noch nie probiert 1	überhaupt nicht 2	nicht sehr einfach 3	relativ einfach 4	sehr einfach 5
Über den Touchscreen durch die Menüs auf dem Bildschirm navigieren.	☐	☐	☐	☐	☐
Die Tastatur auf dem Bildschirm zum Tippen verwenden	☐	☐	☐	☐	☐

2. Kommunikation

Bei der Nutzung eines mobilen Endgeräts kann ich:	noch nie probiert 1	überhaupt nicht 2	nicht sehr einfach 3	relativ einfach 4	sehr einfach 5
E-Mails senden	☐	☐	☐	☐	☐
Bilder per E-Mail senden	☐	☐	☐	☐	☐

3. Datenspeicherung

Bei der Nutzung eines mobilen Endgeräts kann ich:	noch nie probiert 1	überhaupt nicht 2	nicht sehr einfach 3	relativ einfach 4	sehr einfach 5
Informationen (Dateien wie Musik, Bilder, Dokumente) von meinem mobilen Gerät <u>auf</u> meinen Computer übertragen.	☐	☐	☐	☐	☐
Informationen (Dateien wie Musik, Bilder, Dokumente) von meinem Computer <u>auf</u> mein mobiles Gerät übertragen.	☐	☐	☐	☐	☐

4. Internet

Bei der Nutzung eines mobilen Endgeräts kann ich:	noch nie probiert 1	überhaupt nicht 2	nicht sehr einfach 3	relativ einfach 4	sehr einfach 5
Informationen zu meinen Hobbies und Interessen im Internet finden	☐	☐	☐	☐	☐
Informationen zu Gesundheit im Internet finden	☐	☐	☐	☐	☐

5. Kalender

Bei der Nutzung eines mobilen Endgeräts kann ich:	noch nie probiert 1	überhaupt nicht 2	nicht sehr einfach 3	relativ einfach 4	sehr einfach 5
Veranstaltungen und Verabredungen in einen Kalender eintragen	☐	☐	☐	☐	☐
Das Datum und die Uhrzeit von anstehenden und vergangenen Verabredungen nachschauen	☐	☐	☐	☐	☐

6. Unterhaltung

Bei der Nutzung eines mobilen Endgeräts kann ich:	noch nie probiert 1	überhaupt nicht 2	nicht sehr einfach 3	relativ einfach 4	sehr einfach 5
Den "Online-Shop" des Geräts nutzen, um Spiele und andere Formen der Unterhaltung zu finden (z.B. im Apple App Store oder im Google Play Store)	☐	☐	☐	☐	☐
Musik hören	☐	☐	☐	☐	☐

7. Privatsphäre

Bei der Nutzung eines mobilen Endgeräts kann ich:	noch nie probiert 1	überhaupt nicht 2	nicht sehr einfach 3	relativ einfach 4	sehr einfach 5
Ein Passwort vergeben, um das Gerät zu sperren und entsperren	☐	☐	☐	☐	☐
Den Verlauf des Internetbrowsers und alle temporären Daten löschen.	☐	☐	☐	☐	☐

8. Fehlerbehebung und Umgang mit Programmen

Bei der Nutzung eines mobilen Endgeräts kann ich:	noch nie probiert 1	überhaupt nicht 2	nicht sehr einfach 3	relativ einfach 4	sehr einfach 5
Updates für Spiele und andere Apps installieren	☐	☐	☐	☐	☐
Spiele und andere Apps löschen.	☐	☐	☐	☐	☐
