# Supplementary material for: Toward harmonization of aging and technology research: German adaptation of the mobile device proficiency questionnaire (MDPQ) for older adults
Source: Eur J Ageing. 2024 Dec 1;21(1):38. doi: 10.1007/s10433-024-00834-w (PMC11609131; doi:10.1007/s10433-024-00834-w)
Supplement: Supplementary file 2 — Supplementary file2 (PDF 427 kb) [file 10433_2024_834_MOESM2_ESM.pdf]

Supplement B

Table I. Intercorrelations of MDPQ-16G items in study 1 (full sample)

MDPQ-16G items	1b	2a	2b	3a	3b	4a	4b	5a	5b	6a	6b	7a	7b	8a	8b
1a: Navigate using touchscreen	**.64**	.53	.53	.52	.47	.54	.54	.54	.53	.56	.57	.54	.52	.63	.64
1b: Use keyboard		.56	.49	.45	.37	.60	.58	.42	.43	.47	.45	.49	.43	.49	.49
2a: Send emails			**.75**	.52	.46	.61	.59	.45	.45	.49	.47	.47	.42	.48	.49
2b: Send pictures by email				.64	.57	.55	.53	.50	.49	.53	.50	.53	.53	.54	.57
3a: Transfer to computer					**.83**	.47	.44	.51	.50	.53	.57	.56	.59	.58	.60
3b: Transfer from computer						.41	.39	.51	.50	.54	.58	.54	.59	.57	.59
4a: Find information about hobbies							**.90**	.42	.42	.51	.52	.53	.47	.55	.55
4b: Find health information								.40	.40	.50	.50	.51	.46	.50	.50
5a: Enter events into calendar									**.93**	.56	.52	.52	.50	.52	.55
5b: Check calendar										.55	.51	.49	.47	.51	.54
6a: Find entertainment apps											**.68**	.58	.58	.68	.71
6b: Listen to music												.56	.60	.59	.63
7a: Setup password													**.63**	.59	.62
7b: Erase temporary files														.61	.66
8a: Update apps															**.88**
8b: Delete apps															-

Note: Spearman correlations; all correlations are significant at $p < .001$; correlations of items that belong to the same subscale are printed in bold.

Table II. Intercorrelations of MDPQ-16G items in study 2 (non-panel participants)

MDPQ-16G items	1b	2a	2b	3a	3b	4a	4b	5a	5b	6a	6b	7a	7b	8a	8b
1a: Navigate using touchscreen	**.51**	.34	.38	.46	.45	.50	.47	.40	.41	.50	.38	.53	.38	.47	.44
1b: Use keyboard		.36	.31	.31	.33	.41	.36	.27	.31	.34	.28	.36	.31	.32	.34
2a: Send emails			**.63**	.35	.33	.37	.29	.25	.31	.33	.29	.25	.30	.29	.35
2b: Send pictures by email				.47	.42	.41	.32	.41	.46	.46	.41	.28	.34	.44	.48
3a: Transfer to computer					**.83**	.47	.38	.48	.52	.48	.45	.50	.54	.60	.56
3b: Transfer from computer						.42	.39	.40	.47	.43	.48	.48	.54	.57	.54
4a: Find information about hobbies							**.84**	.37	.39	.46	.41	.47	.44	.43	.41
4b: Find health information								.34	.37	.41	.42	.47	.44	.41	.37
5a: Enter events into calendar									**.87**	.56	.45	.55	.48	.60	.54
5b: Check calendar										.55	.49	.51	.52	.59	.56
6a: Find entertainment apps											**.55**	.60	.51	.65	.67
6b: Listen to music												.49	.50	.53	.53
7a: Setup password													**.60**	.59	.57
7b: Erase temporary files														.53	.54
8a: Update apps															**.80**
8b: Delete apps															-

Note: Spearman correlations; all correlations are significant at $p < .001$; correlations of items that belong to the same subscale are printed in bold.

Table III. MDPQ-16G scores and reliabilities in study 1 (panel participants and non-panel participants)

Scale	*n*	*M*	*SD*	*α*	r_{SB}	r_S
**MDPQ-16G**	**226 (318)**	**27.6 (31.6)*** **	**9.57 (7.90)**	**.95 (.94)**	**-**	**-**
Mobile Device Basics	228 (319)	4.0 (4.3)***	1.20 (0.97)	-	.84 (.81)	.65 (.62)
Communication	228 (319)	4.1 (4.5)***	1.37 (0.90)	-	.90 (.87)	.78 (.72)
Data and File Storage	228 (318)	2.8 (3.5)***	1.48 (1.32)	-	.94 (.86)	.88 (.78)
Internet	228 (318)	4.1 (4.4)***	1.34 (0.98)	-	.97 (.91)	.95 (.86)
Calendar	228 (318)	3.1 (3.8)***	1.73 (1.63)	-	.97 (.97)	.92 (.92)
Entertainment	226 (318)	3.1 (3.5)**	1.58 (1.55)	-	.74 (.83)	.61 (.72)
Privacy	226 (318)	3.3 (3.8)***	1.42 (1.28)	-	.72 (.80)	.57 (.66)
Troubleshooting and Software Management	226 (318)	3.1 (3.8)***	1.61 (1.46)	-	.94 (.94)	.88 (.88)

Note: MDPQ-16G = German short version of Mobile Device Proficiency Questionnaire; *M* = mean; *SD* = standard deviation; α = Cronbach´s α; ω = McDonald's ω; r_{SB} = Spearman-Brown reliability estimate; r_S = Spearman correlations between two items of the subscales, all significant at p < .001; possible range of MDPQ-16G = 8-40; possible range of subscales = 1-5; α and ω can only be computed if the number of items is > 2; first value presents results for panel participants; results for non-panel participants are provided in brackets; differences between panel and non-panel participants were tested using t-tests for independent samples, *** : difference is significant at $p < .001$, **: difference is significant at $p < .01$.

Table IV. Validity related findings of the MDPQ-16G in study 1 (panel participants and non-panel participants)

***t*-Tests for independent samples**					
	***M* male**	***SD* male**	***M* female**	***SD* female**	***t***
Panel	30.0	9.18	26.5	9.56	$t(134) = 2.64$, $p = .009$, d = .38
Non-Panel	32.9	8.20	30.2	8.20	$t(316) = 3.11$, $p = .002$, d = .35
	***M* education low**	***SD* education low**	***M* education high**	***SD* education high**	
Panel	26.0	9.88	28.3	9.35	$t(224) = 1.75$, $p = .081$, d = .25
Non-Panel	31.3	7.92	31.7	7.88	$t(312) = 0.35$, $p = .730$, d = .05
**Correlation Analyses with Age and Technology Device Use**					
	**Age**	**Smartphone Use**	**Tablet Use**	**PC Use**	
Panel	$r = -.33$, $p < .001$	$r =.62$, $p < .001$	$r =.41$, $p < .001$	$r =.20$, $p = .003$	
Non-Panel	$r = -.35$, $p < .001$	$r =.51$, $p < .001$	$r =.40$, $p < .001$	$r =.00$, $p = .967$	
**Correlation Analyses with and Technology Attitudes**					
	**TAM usefulness**	**TAM ease of use**	**STAI**		
Panel	$r = .44$, $p < .001$	$r = .38$, $p < .001$	$r = .49$, $p < .001$		
Non-Panel	$r = .29$, $p < .001$	$r = .48$, $p < .001$	$r = .56$, $p < .001$		

Note: MDPQ-16G = short version of Mobile Device Proficiency Questionnaire; *M* = mean; *SD* = standard deviation; *r* = Pearson correlations, TAM = Technology Acceptance Model; STAI = Subjective Technology Adaptivity Inventory; Education: low = no University entrance qualification / high = university entrance qualification; possible range of MDPQ-16G = 8-40.
